# Developmental and epileptic encephalopathy 44 due to compound heterozygous variants in the *UBA5* gene: a case report

**DOI:** 10.1186/s42494-023-00139-y

**Published:** 2023-11-13

**Authors:** Suli Zhang, Shuangzhu Lin, Wanqi Wang, Yuru Gan, Cui Wang, Bangtao Li, Qiming Pang

**Affiliations:** 1 Department of Neuroscience, Hainan Women and Children’s Medical Center, Haikou, 570100 China; 2 Diagnosis and Treatment Center for Children, First Affiliated Hospitalto , Changchun University of Chinese Medicine, Changchun, 130021 China; 3 Changchun University of Chinese Medicine, Changchun University of Chinese Medicine Graduate School, Changchun, 130000 China

**Keywords:** Infantile spasms, Epilepsy, Developmental and epileptic encephalopathy, *UBA5* gene

## Abstract

**Background:**

Developmental and epileptic encephalopathy (DEE) is a group of rare inherited disorders characterized by intellectual disability, delayed development, epileptic seizures, and other related symptoms. DEE44 is caused by mutations in the *UBA5* gene, which encodes a ubiquitin-like protein involved in protein degradation and cell signaling. However, there is limited information on the genotype–phenotype correlation of DEE44, and its clinical features remain to be fully characterized.

**Case presentation:**

We report a 12-month-old infant who presented with epileptic spastic seizures beginning at 4 months of age, accompanied by overall developmental delay, short stature, microcephaly, inability to hold his head upright, chasing vision, and high muscle tone in the extremities. Genetic findings showed compound heterozygous mutations of the *UBA5* gene: NM_024818 c.562C > T(p.R188X) from the mother and NM_024818 c.214C > T(p.R72C) from the father.

**Conclusions:**

This case report expands the clinical spectrum of DEE44 and highlights the importance of considering DEE44 in the differential diagnosis of developmental delay and epilepsy, even in the absence of classical symptoms suggestive of the condition. We hope that this case report will advance the understanding of DEE44 and improve the expertise of clinicians and early diagnose of this disease.

## Background

Developmental and epileptic encephalopathy (DEE) 44 is an autosomal recessive neurological disorder caused by mutations in the *UBA5* gene. The disease onset usually occurs within weeks or months after birth, with a variety of clinical signs and symptoms. The most common clinical manifestations are spasmodic seizures, global developmental delay, and electroencephalogram (EEG) hyperarrhythmia, consistent with clinical diagnosis of West syndrome. Most patients have poor prognosis, and some die in childhood [1, 2].

## Case presentation

A 12-month-old male patient had convulsions without obvious causes at age of < 1 month. He showed spasmodic-tonic seizures with specific symptoms including staring of both eyes to one side, holding hands in front of the chest, head tilting to the right, sometimes accompanied by raising up of upper and lower limbs, rapid shaking of limbs, and crying. During epileptic spasms (sometimes occur in clusters), the child held his hands in front of his chest, his head deflected to the right, with or without upper and lower limb elevation, and his limbs shaked rapidly.

The attacks occurred during both awake and sleeping periods, more commonly during the former. After admission, a detailed consultation, physical examination, and comprehensive laboratory examination were conducted for the patient.

The patient’s parents reported no previous history of special diseases, infection, surgical trauma, food or drug allergies, or blood transfusions. The child was delivered by his mother (G1P1) spontaneously at 39 weeks with a birth weight of 2.75 kg and a birth height of 49 cm. There was no history of asphyxia or rescue at birth. The parents were in good health. They reported no intermarriage in the child’s family history and that the family did not have any history of genetic diseases. However, we noticed that the child had clear growth and developmental retardation. At the age of 1 year, the child could not stand up, tended to stare with poor fixation, could not grasp objects, and occasionally laughed but could not make “babbling” sounds. Physical examination showed a height of 61 cm (- 3 SD), weight of 5.8 kg (- 3 SD), and head circumference of 38 cm. In addition, the child was unable to raise his head or turn over on his own; he had a high palatine arch, palmar crease on the right hand and congenital laryngeal cartilage softening, with increased muscle tension and limb muscle strength of grade. During a vision examination, the child was able to follow light and objects, was sensitive to light reflection, and demonstrated a soft neck without resistance. He didn't show any pathological signs. Other cardiopulmonary and abdominal examinations also showed no obvious abnormalities.

**Fig. 1:**
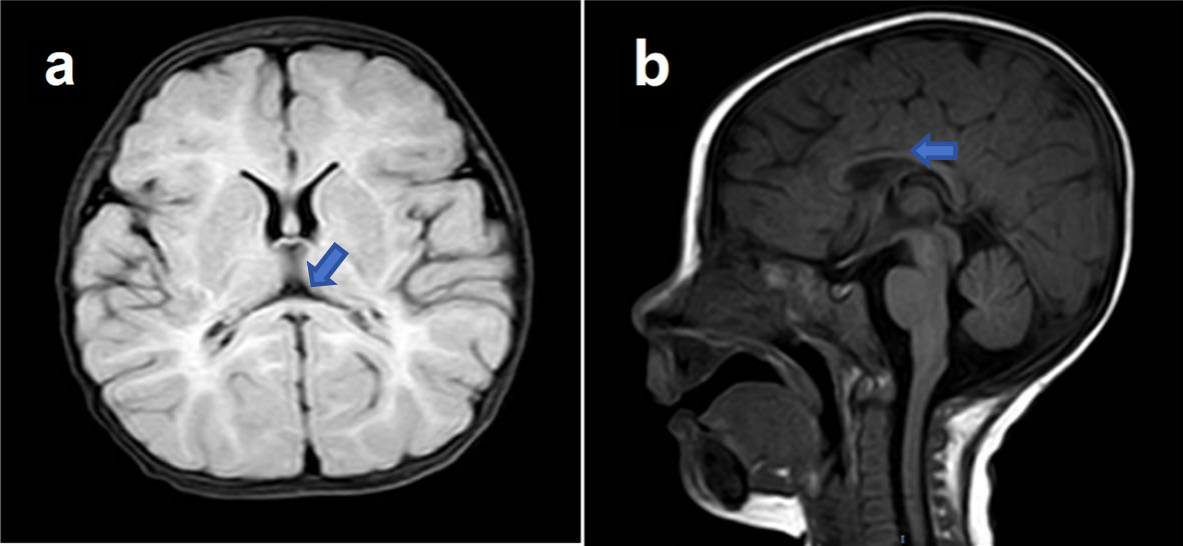
MRI showed weak white matter myelination signals of the corpus callosum (**a**, **b**)

A series of comprehensive laboratory examinations was conducted, including routine blood tests, urine tests, stool tests, liver and kidney function tests, myocardial enzymes tests, electrolytes tests, urine tandem mass spectrometry, blood tandem mass spectrometry, as well as tests for ceruloplasmin levels, homocysteine levels, parathyroid hormone levels, and blood ammonia levels. The above examinations had normal results. We further performed brain magnetic resonance imaging (MRI) and video EEG. MRI showed thinning of the corpus callosum (Fig. 1a, b). while the cerebellomedullary cistern was relatively full.

**Fig. 2:**
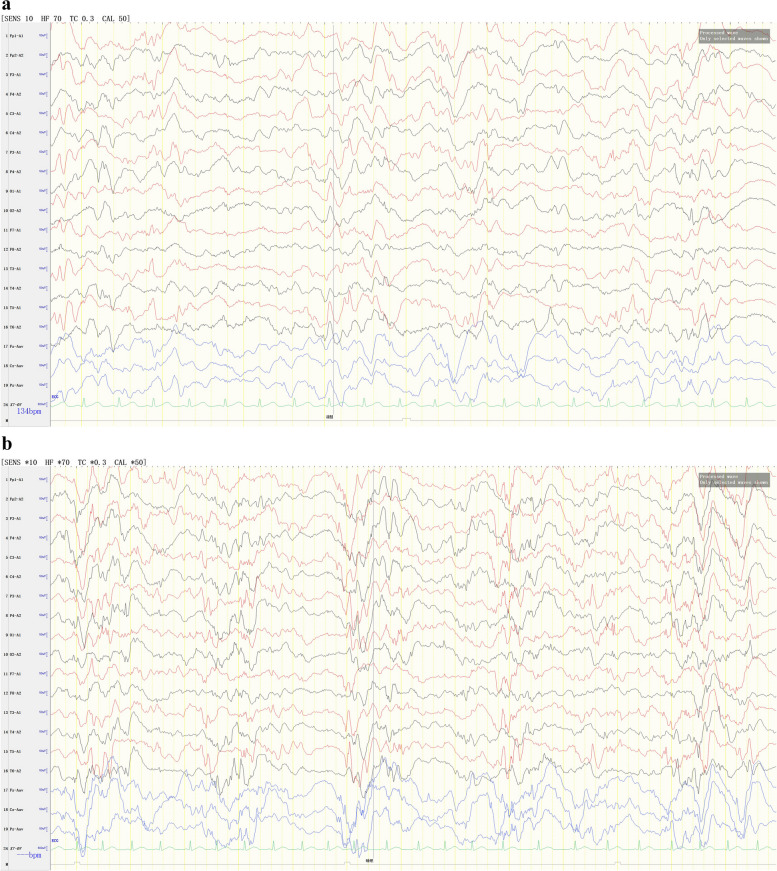
During wake (a) and sleep periods (b) of the interictal phase, abnormal waves were observed, including a large number of high-amplitude slow waves, sharp waves, spike waves, spike-wave rhythms, and spike-slow waves, showing multifocal and generalized continuous distribution and partial hypsarrhythmia

**Fig. 3:**
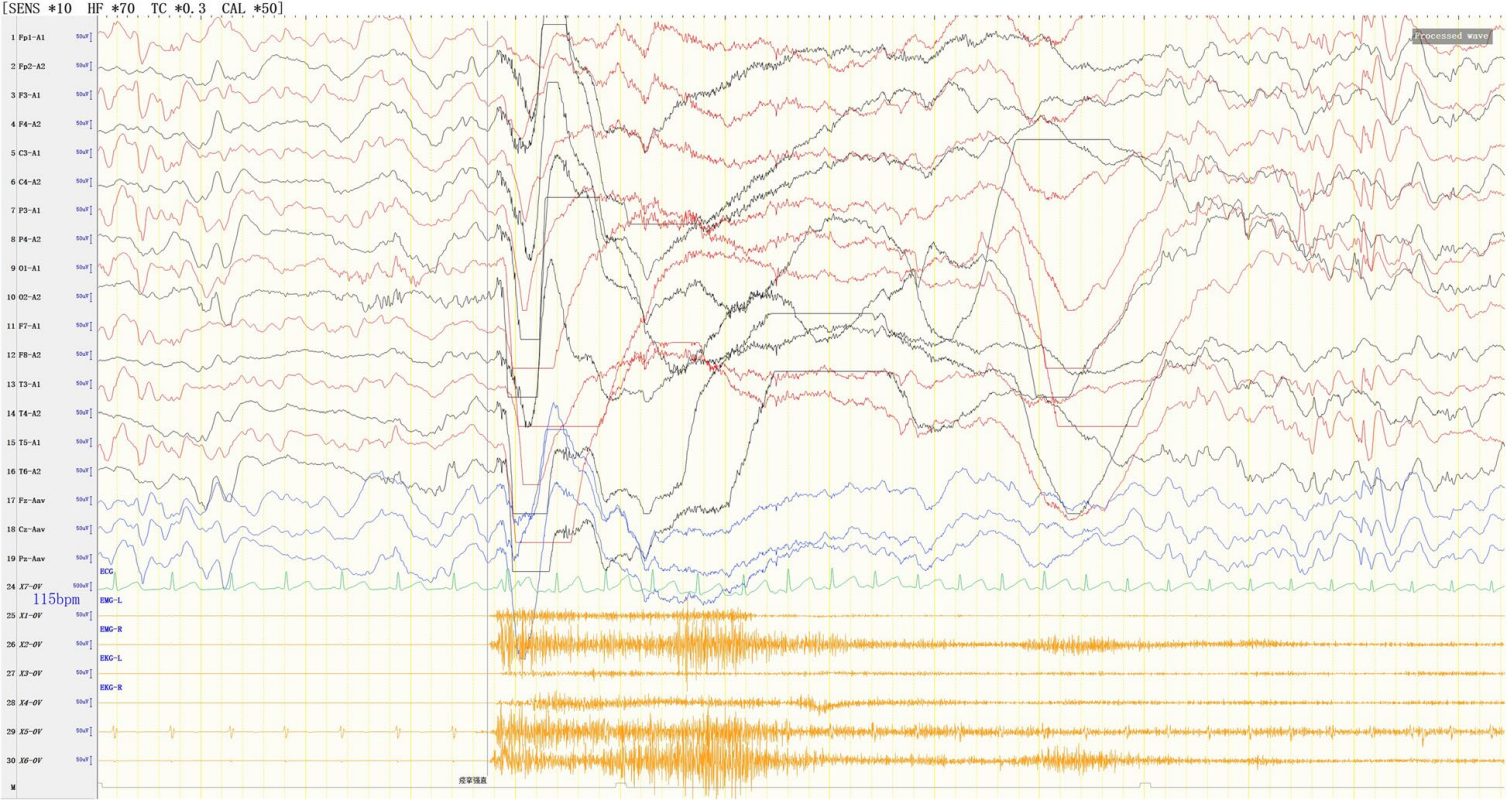
Spasmodic-tonic seizures. Electromyography (EMG) showed continuous electromyographic burst

**Fig. 4:**
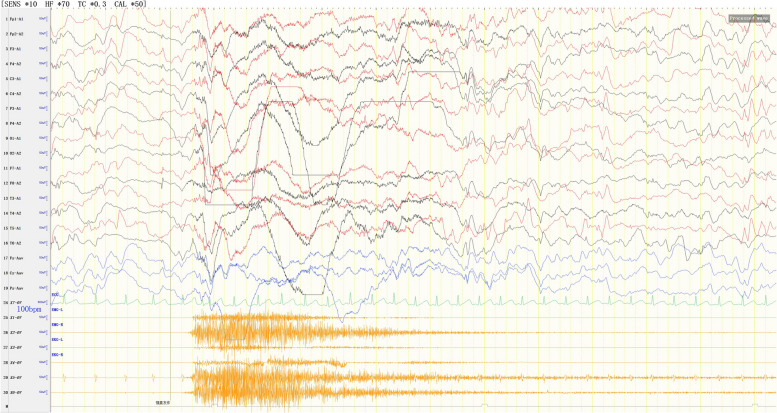
Tonic seizures. EEG showed rapid, generalized low-amplitude wave rhythms when the child was displaying straight, stiff limbs and was crying. EMG showed continuous electromyographic bursts

**Fig. 5:**
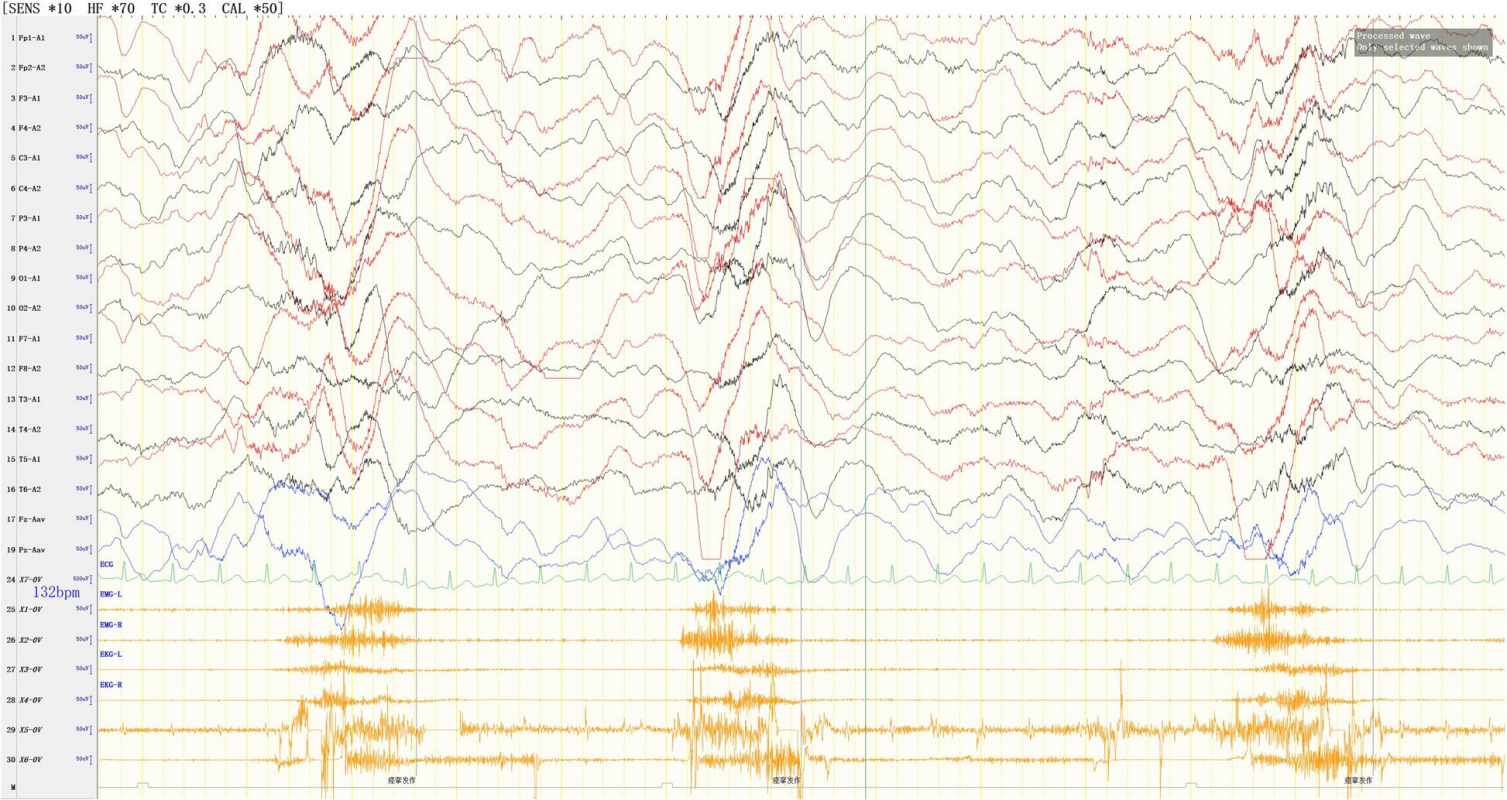
Epileptic spasms (sometimes occuring in clusters). EEG showed generalized, high-amplitude slow waves accompanied by a low-amplitude spike rhythm release, and EMG showed an electromyographic diamond-shaped emission at the same time

Video EEG demonstrated the following findings: (1) EEG background: no dominant rhythms in the occipital region; (2) multifocal and generalized epileptiform discharges throughout the entire brain region (Fig. 2 ); highly disorganized activities during wake and sleep periods (Fig. 2), (3) occurrence of multiple spasm attacks, in addition to tonic–clonic seizures (Fig. 3), tonic seizures (Fig. 4), and epileptic spasms (sometimes occuring in clusters) (Fig. 5).

**Fig. 6:**
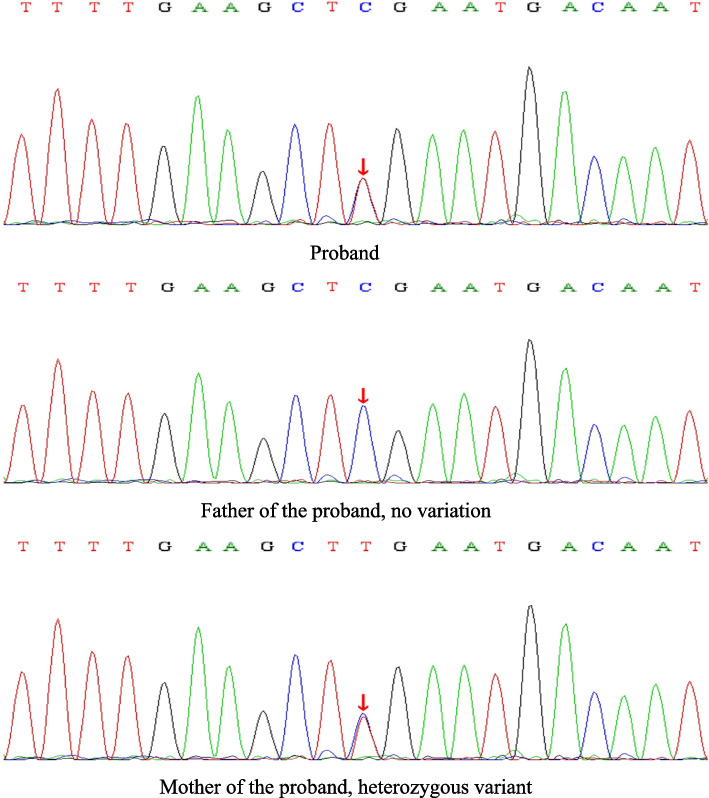
Sanger sequencing results of *UBA5*: NM_024818: exon6: c.562C > T(p.R188X)

**Fig. 7:**
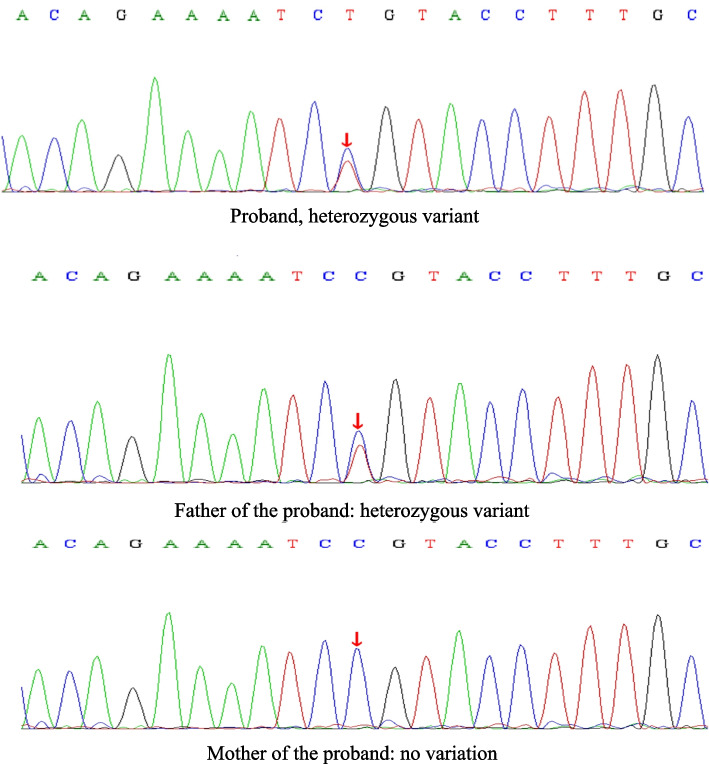
Sanger sequencing results of *UBA5*: NM_024818: exon3: c.214C > T (p.R72C)

The onset of the disease occurred at a young age, presenting with evident epilepsy symptoms, together with obvious abnormalities from head MRI examination and video EEG. Therefore, we highly suspected that the disease was attributed to genetic mutations, and an exome sequencing analysis was performed for the entire family of the patient. With the duly obtained informed consent from the child’s parents, peripheral blood was collected from the child and his parents for the whole-exome genetic sequencing (Beijing MyGenostics). The results of Sanger sequencing revealed that the child carried the complex heterozygous mutation of *UBA5* c.562C > T(p.R188X). This mutation was derived from his parents. His mother had a heterozygous mutation, NM_024818: exon6: c.562C > T(p.R188X), while his father had no variants (Fig. 6). Interestingly, his father had a heterozygous mutation, NM_024818: exon3: c.214C > T(p.R72C), while his mother had no variants (Fig. 7). The child was finally diagnosed as DEE44 caused by *UBA5* gene mutation. Concurrent with genetic testing, proactive treatment strategies were implemented, including administration of adrenocorticotropic hormone (ACTH) and magnesium sulfate shock therapy. Regrettably, the therapeutic outcome proved to be unsatisfactory, as the child continued to experience several seizures daily.

## Discussion

The *UBA5* gene is located at chromosome 3q22.1 (OMIM: 610552), encoding ubiquitin-like modifier activating enzyme 5 (UBA5). The UBA5 protein is a member of the E1-like ubiquitin activator family, which activates ubiquitin-folding modifier 1, a ubiquitin-like posttranslational modifier protein, by forming high-energy thioester bonds [3]. Animal studies have highlighted the importance of UBA5 in erythrocyte development, indicating its involvement in the hematopoietic process [4]. Moreover, UBA5 has been identified as an essential player in the development of the nervous system. Mutations in the *UBA5* gene have been found to be responsible for a range of clinical symptoms, such as significant motor deficits, shortened lifespan, and neuromuscular junction defects [1, 2, 5].

Mutations in the *UBA5* gene have been identified as the underlying cause of DEE44 and spinocerebellar ataxia with autosomal recessive 24 (SCAR24), both of which are inherited in an autosomal recessive manner [1, 2, 5]. At present, nearly 30 cases of DEE44 have been reported. Children with DEE resulting from *UBA5* gene mutations exhibit a range of clinical symptoms. The primary symptoms include microcephaly, short stature, visual impairment, mental retardation, motor impairment, and seizures. Secondary symptoms may also manifest, including hereditary cerebellar ataxia, abnormal gait, limb ataxia, dysarthria, horizontal nystagmus, cataracts, and cerebellar atrophy, which may be accompanied by demyelinating peripheral neuropathy [1, 2, 5–12]. SCAR24 is relatively rare in clinical practice. As reported by Duan et al. in 2016, the main clinical manifestations of SCAR24 are gait and limb ataxia, dysarthria, nystagmus, and cataracts [5]. Brain imaging may show the presence of cerebellar atrophy, suggesting its progressive nature [6].

The case reported here had severe seizures, delayed overall development, short stature, and microcephaly. EEG recording showed hypsarrhythmia consistent with typical DEE44 manifestations. The *UBA5* gene in this patient contained two mutations, c.562C > T(p.R188X) derived from his mother and c.214C > T (p.R72C) from his father. According to the guideline of the American College of Medical Genetics and Genomics (ACMG), the c.562C > T(p.R188X) variant is determined to be a pathogenic mutation and there are several case reports of recessive inheritance at this site [2]. The other mutation c.214C > T (p.R72C) remains unclear for its clinical significance according to ACMG guidelines, although there are reports of recessive genetic cases at this locus [6]. Based on the clinical presentation and the identified genetic mutation, we ruled out the possibility of SCAR24 and finally arrived at the diagnosis of DEE44.

Regrettably, there is no specific treatment for DEE44 at present. We administered a combined ACTH and magnesium sulfate treatment as a shock therapy based on the symptoms, but did not observe satisfactory clinical efficacy, as the child continued to experience several seizures every day. Additionally, the child was also treated with several anti-seizure medications, including valproate, topiramate, perampanel, vigabatrin, and clobazam. Despite these interventions, the child died of type I respiratory failure at the age of 1 year and 4 months.

## Conclusions

We report a case of developmental epileptic encephalopathy induced by a compound heterozygous variant of the *UBA5* gene. This report extends the understanding of the phenotypic and genetic variations associated with this disorder, and offer insights and guidance for clinicians in the diagnosis and management of similar cases.

## Data Availability

The data presented in this study are available on request from the corresponding author.

## Abbreviations

ACTH: Adrenocorticotropic hormone
ACMG: The American College of Medical Genetics and Genomics
DEE: Developmental and epileptic encephalopathy
EEG: Electroencephalogram
EMG: Electromyography
MRI: Magnetic resonance imaging
SCAR24: Spinocerebellar ataxia with autosomal recessive 24
